# Using Immune-Related Long Non-coding Ribonucleic Acids to Develop a Novel Prognosis Signature and Predict the Immune Landscape of Colon Cancer

**DOI:** 10.3389/fcell.2021.750709

**Published:** 2021-09-30

**Authors:** Xu Wang, Ke Chen, Zhenglin Wang, Yuanmin Xu, Longfei Dai, Tao Bai, Bo Chen, Wenqi Yang, Wei Chen

**Affiliations:** Department of General Surgery, The First Affiliated Hospital of Anhui Medical University, Hefei, China

**Keywords:** immune related long noncoding RNAs, prognosis signature, colon cancer, tumor-infiltrating immune cell, immune checkpoint genes, chemotherapeutics

## Abstract

**Purpose:** This study aimed to construct a novel signature to predict the survival of patients with colon cancer and the associated immune landscape, based on immune-related long noncoding ribonucleic acids (irlncRNAs).

**Methods:** Expression profiles of irlncRNAs in 457 patients with colon cancer were retrieved from the TCGA database (https://portal.gdc.cancer.gov). Differentially expressed (DE) irlncRNAs were identified and irlncRNA pairs were recognized using Lasso regression and Cox regression analyses. Akaike information criterion (AIC) values of receiver operating characteristic (ROC) curve were calculated to identify the ideal cut-off point for dividing patients into two groups and constructing the prognosis signature. Quantitative real-time polymerase chain reaction (qRT-PCR) was performed to validate the expression of LINC02195 and SCARNA9 in colon cancer.

**Results:** We identified 22 irlncRNA pairs and patients were divided into high-risk and low-risk groups based on the calculated risk score using these 22 irlncRNA pairs. The irlncRNA pairs were significantly related to patient survival. Low-risk patients had a significantly longer survival time than high-risk patients (*p* < 0.001). The area under the curve of the signature to predict 5-year survival was 0.951. The risk score correlated with tumor stage, infiltration depth, lymph node metastasis, and distant metastasis. The risk score remained significant after univariate and multivariate Cox regression analyses. A nomogram model to predict patient survival was developed based on the results of Cox regression analysis. Immune cell infiltration status, expression of some immune checkpoint genes, and sensitivity to chemotherapeutics were also related to the risk score. The results of qRT-PCR revealed that LINC02195 and SCARNA9 were significantly upregulated in colon cancer tissues.

**Conclusion:** The constructed prognosis signature showed remarkable efficiency in predicting patient survival, immune cell infiltration status, expression of immune checkpoint genes, and sensitivity to chemotherapeutics.

## Introduction

Despite the rapid development of medical treatments, the trends in cancer incidence and death rates have been increasing worldwide. Specifically, the incidence and mortality rate of colon cancer are relatively high (Bray et al., 2018). Patients with early colon cancer can be treated by surgery; however, most patients with advanced colon cancer experience cancer recurrence and metastasis, and their 5-year survival rate is lower than 10% (Bhandari et al., 2017; Doonan et al., 2017; Russo et al., 2019). With the development of chemotherapy and targeted medicine, the overall survival rate of patients with colon cancer is significantly higher now than before. In recent years, advances in tumor immune therapy and application of immune checkpoint inhibitors have led to improvements in cancer treatment.

Programmed cell death protein 1 (PD-1), first discovered in 1992, is a 288 amino acid protein expressed on the surface of T cells and related to apoptosis (Ishida et al., 1992). When PD-1 is bound to its ligand programmed cell death ligand 1 (PD-L1), the anti-tumor effect of T cells is inhibited (Kouo et al., 2015). PD-1-blocking antibodies, pembrolizumab and nivolumab, were approved by the United States Food and Drug Administration (FDA) for the treatment of refractory melanoma and advanced non-small cell lung cancer in 2014 and 2015, respectively. Atezolizulab, the first anti-PD-L1 antibody, was approved for the treatment of urothelial cancers in 2016. With the rapid development of tumor immunotherapy, several immune checkpoint inhibitors have been used in the treatment of various types of malignant tumors. According to a recent meta-analysis (He et al., 2020), anti-PD-1 inhibitors have high efficacy and have led to a better prognosis in patients with deficient mismatch repair (dMMR)/microsatellite instability high (MSI-H) metastatic colorectal cancer (mCRC) (dMMR/MSI-H mCRC).

Long non-coding ribonucleic acids (lncRNAs), defined as RNAs longer than 200 nucleotides, are not translated into functional proteins (Iyer et al., 2015). The completely spliced lncRNA is transported into the cytoplasm or other organelles through a mechanism similar to that of mRNA. Once in the cytoplasm, lncRNAs transregulate gene expression at the post-transcriptional level, such as regulating mRNA translation and degradation, or participating in the regulation of intracellular signaling pathways (Statello et al., 2021). Recent studies have shown that lncRNAs not only change the genome or transcriptome, but also modify the immune microenvironment to contribute to the malignant phenotype of cancer. LncRNAs direct the expression of genes related to immune cell activation, leading to tumor immune cell infiltration (Atianand et al., 2017; Chen et al., 2017). Several lncRNAs are differentially expressed (DE) in various types of tumor tissues (Schmitt and Chang, 2016).

In recent years, many researchers have focused on constructing signatures using lncRNAs for predicting prognosis of patients with cancer based on the cancer Genome Atlas (TCGA) database. Qi et al. (2021) identified eight immune-related (ir) lncRNAs and developed a signature for the prognosis of patients with pancreatic adenocarcinoma. Zhou et al. (2021) identified an irlncRNA signature to predict the prognosis, immune cell infiltration, and immunotherapy response in patients with hepatocellular carcinoma and validated the expression of the six lncRNAs using the quantitative real-time polymerase chain reaction (qRT-PCR) method. Zhang et al. (2021) built a signature based on ten hypoxia-related lncRNAs that showed promising predictive effect for patient prognosis. Ma et al. (2021) identified a metabolism-related lncRNA signature for prediction of risk of recurrence in patients with breast cancer. Compared with using single biomarkers, combinations of two biomarkers lead to much more accurate diagnostic models for cancers (Lv et al., 2020). Moreover, these combinations do not require any specific expression levels or testing methods. However, not many studies have focused on the development of signatures based on lncRNA pairs for diagnosis or survival prediction in patients with colon cancer. We constructed a prognosis signature using irlncRNA pairs, which showed remarkable efficiency in predicting patient survival, immune cell infiltration status, expression of immune checkpoint genes, and sensitivity to chemotherapeutics.

## Materials and Methods

### Immune-Related Long Noncoding Ribonucleic Acids Identification and Expression

Expression patterns of irlncRNAs of patients with colon cancer were downloaded from the Genomic Data Commons (GDC) Data Portal.^1^ LncRNAs and mRNAs were distinguished using GTF files, which were downloaded from Ensembl.^2^ A list of human immune-related genes was prepared using the ImmPort database,^3^ a publicly available repository containing up-to-date information on human genes and proteins that are involved in immunity. Co-expression analysis was performed between immune-related genes and all lncRNAs to identify irlncRNAs. The screening criteria used was correlation coefficient >0.4 and *p*-value < 0.001. *limma* package of R software (version 4.0.3) was used to screen out differentially expressed immune-related lncRNAs (DEirlncRNAs) with criteria of log | fold change (FC)| >2.5 and *p*-value < 0.01.

### Gene Oncology and Kyoto Encyclopedia of Genes and Genomes Analyses

Pearson correlation coefficient analysis was performed to analyze the relationship between the expression of DEirlncRNAs and mRNAs and the top 10 mRNAs were considered to be associated with lncRNAs. To better understand the biological functions and pathways involved in DEirlncRNAs, Gene Oncology (GO), and Kyoto Encyclopedia of Genes and Genomes (KEGG) analyses were performed using *ggplot2*, *Bioconductor*, and *org.Hs.eg.db* R packages. *P*-values and *q*-values < 0.05 were considered statistically significant.

### Construction of Differentially Expressed Immune-Related Long Non-coding Ribonucleic Acid Pairs

A DEirlncRNA pair was constructed using two DEirlncRNAs, for example, lncRNA A and lncRNA B. A 0-or-1 matrix was constructed. If the ratio of lncRNA A to lncRNA B was higher than 1, the expression of the lncRNA pair was defined as 1, otherwise, it was defined as 0. If the proportion of lncRNA pairs with expression defined as 0 or 1 was less than 20% or more than 80%, the pair was considered invalid.

### Clinical Data of Colon Cancer Patients

Clinical information of patients with colon cancer was downloaded from colon adenocarcinoma (COAD) project of TCGA database. Patients with no follow-up or incomplete clinical information were excluded. After selection, 393 cases of colon cancer were included in this study.

### Development and Validation of the Prognosis Signature Using Differentially Expressed Immune-Related Long Non-coding Ribonucleic Acid Pairs

Least Absolute Shrinkage and Selection Operator (LASSO) regression analysis and Cox regression analysis were performed to screen out prognosis-related lncRNA pairs using *survival*, *survminer*, and *glmnet* R packages. LncRNA pairs with *p* < 0.05 were considered significant. After selection, 22 lncRNA pairs were included to construct the prognosis signature. Risk scores were calculated based on the following formula: Risk score = $\sum_{i=1}^{\text{n}}\beta \,i^{*}\,\lambda \,i$, where n represents the numbers of lncRNA pairs included to construct the signature and β*i* and λ*i* represent the regression coefficient and expression value of lncRNA pairs, respectively. According to the maximum akaike information criterion (AIC) value of 5-year receiver operating characteristic (ROC) curve, patients were divided into high-risk and low-risk groups. ROC and AUC were used to test the prediction efficiency and compare the constructed signature and other clinical variables. The Kaplan-Meier (KM) method and log rank test were used to evaluate survival differences between high-risk and low-risk groups. Univariate and multivariate analyses were used to determine whether risk score was an independent predictor of prognosis in patients with colon cancer. Chi-square test was performed to analyze the relationship between the signature and clinical variables and the Wilcoxon signed-rank test was used to show the risk score differences between different groups for these clinical characteristics. A nomogram model was developed based on three independent prognosis factors that were significant in both the univariate and multivariate analyses (*p* < 0.05). Calibration graphs were constructed to show the differences between nomogram-predicted and actual survival rates of patients with colon cancer.

### Immune Cell Infiltration Analysis

To better understand the relationship between the calculated risk score and tumor immune cell infiltration status, datasets including XCELL, TIMER, QUANTISEQ, EPIC, CIBERSORT-ABS, and CIBERSORT were used to analyze the immune cell infiltration status. The lollipop diagram was drawn to show the correlation between risk score and immune infiltrated cells *via* Spearman correlation method. The differences of immune cell content in high-risk and low-risk groups were shown as boxplots using Wilcoxon signed-rank test.

### Expression of Immune Checkpoint Genes in High-Risk and Low-Risk Groups

To understand the differences in the expression levels of immune checkpoint genes in the high-risk and low-risk groups, six immune checkpoint genes were selected, including CTLA4, HAVCR2, IDO1, lymphocyte-activation gene 3 (LAG3), PD-1, and PD-L1. Violin plots were drawn to show the results using *ggpubr* R package.

### Evaluating the Differences in Chemosensitivity Between COAD Patients in High-Risk and Low-Risk Groups

IC50, half of the maximum inhibitory concentration, represents the concentration of drug required for 50% inhibition of cancer cells. IC50 was calculated to evaluate the significance of lncRNA-based signature in six types of chemotherapeutic drugs, including camptothecin, doxorubicin, erlotinib, gemcitabine, paclitaxel, and rapamycin, which have been used in the treatment of patients with colon cancer. Wilcoxon signed-rank test was performed to analyze the differences in IC50 in high-risk and low-risk groups. The results are shown as boxplots using *ggpubr*, *pRRophetic*, and *ggplot2* R packages.

### The Verification of LINC02195 and SCARNA9 by Quantitative Real-Time Polymerase Chain Reaction

Ten pairs of colon cancer tissues and adjacent non-cancer tissues were collected from The First Affiliated Hospital of Anhui Medical University, and approved by the Ethics Committee. All participants signed an informed consent form. These samples were collected after surgical resection from colon cancer patients who had never received preoperative chemotherapy or radiotherapy. The HiPure Universal RNA Kit (Shanghai, Magen) was used to extract total RNA from the colon cancer and adjacent non-cancer tissues stored in liquid nitrogen following the manufacturer’s instructions. The concentration and purity of RNA samples was measured using NanoDrop 2000 (Thermo Fisher Scientific, United States). Extracted RNA was reverse transcribed into cDNA using the PrimeScript RT kit (Vazyme, Nanjing, China) according to the protocol. Finally, the concentration of cDNA was measured using TB Green Premix Ex Taq II (GenStar, China) under the LightCycler480 System (Applied Biosystems, Waltham, MA, United States) according to the manufacturer’s instruction. The primer sequences for PCR amplification were as follows: LINC02195, forward: 5′-GTCA CACAGCAAGCCTAAAGAAACG-3′, reverse: 5′-TCAGCCA TAGAGGAGACAGCAAGG-3′; SCARNA9, forward: 5′-AAGG GCATATGTCTGGTGTGTGTG-3′, reverse: 5′-CCCCACCCTC AATCTCATTCATTCC-3′; GAPDH, forward: 5′-GGGAAGG TGAAGGTCGGAGT-3′, reverse: 5′-GGGGTCATTGATGGCA ACA-3′. GAPDH was used as an internal control, and each sample was repeated three times. The relative expression levels of LINC02195 and SCARNA9 were calculated using the 2^–ΔΔ*Ct*^ method. The differences in LINC02195 and SCARNA9 expression between colon cancer tissues and adjacent non-cancer tissues were tested by *t*-tests. The graphs were drawn using GraphPad Prism software (version 9.2.0).

## Results

### Differentially Expressed Immune-Related Long Noncoding Ribonucleic Acids

**FIGURE 1:**
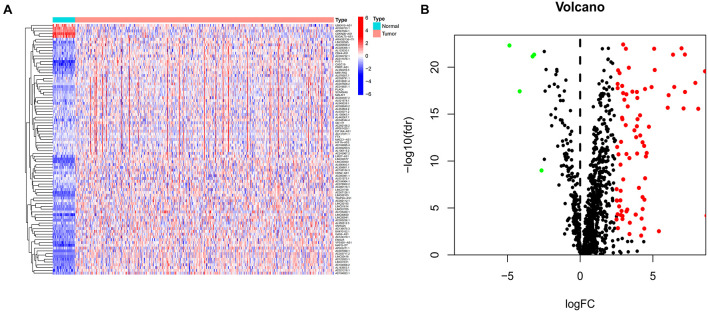
Identification of differentially expressed immune-related lncRNAs (DEirlncRNAs) using TCGA datasets **(A,B)** The heatmap **(A)** and volcano plots **(B)** are shown.

**FIGURE 2:**
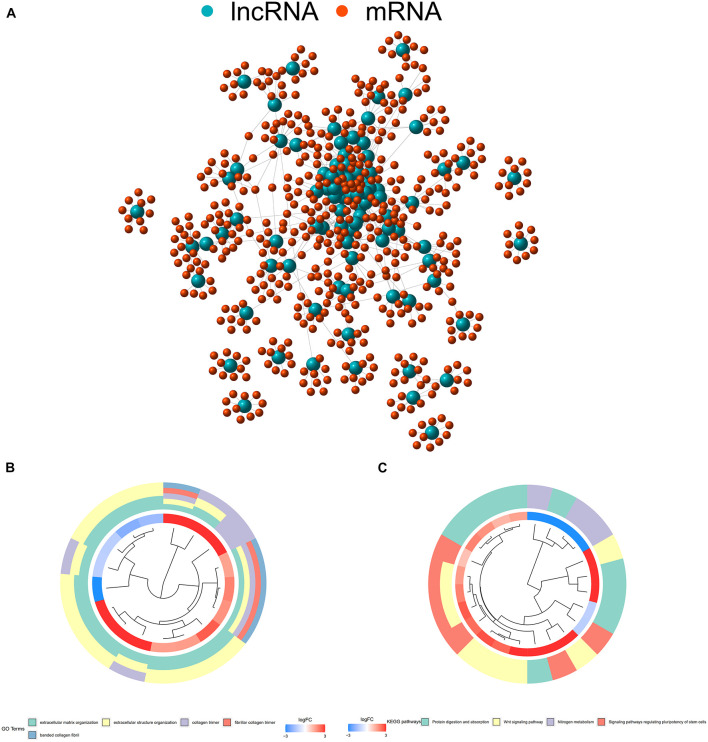
**(A)** The expression correlation between DEirlncRNAs and mRNAs were shown as an Long non-coding ribonucleic acids (lncRNAs)–mRNA co-expression network, wherein lncRNAs and associated mRNAs were linked together with lines. **(B)** DEirlncRNAs were primarily related to the biological functions of extracellular matrix organization, extracellular structure organization, collagen trimers, fibrillar collagen trimers, and banded collagen fibrils. **(C)** These DEirlncRNAs also participated in the pathways of protein digestion and absorption, WNT signaling, nitrogen metabolism, and signaling pathways regulating the pluripotency of stem cells.

Expression patterns and clinical information from 457 COAD patients were downloaded from TCGA database. A total of 393 patients with complete clinical information and follow-up time >0 days were included in the study. Detailed clinical characteristics of the 393 cases are shown in Supplementary Table 1. Human immune-related genes were identified using the ImmPort database. Co-expression analysis was performed to identify irlncRNAs based on immune-related genes. As shown in Figure 1A, there were 90 irlncRNAs with log | FC | >2.5 and *p* value < 0.01, among which 85 lncRNAs were upregulated while five were downregulated (Figure 1B). To understand the biological functions and pathways involved in the 90 DEirlncRNAs, the expression correlation between the DEirlncRNAs and mRNAs were shown as a lncRNA–mRNA co-expression network, and lncRNAs and associated mRNAs were linked together with lines (Figure 2A). As seen in Figure 2B, the 90 lncRNAs were primarily related to the biological functions of extracellular matrix organization, extracellular structure organization, collagen trimer, fibrillar collagen trimer, and banded collagen fibril (Figure 2B). These DEirlncRNAs also participated in the pathways of protein digestion and absorption, WNT signaling, nitrogen metabolism, and regulating pluripotency of stem cells (Figure 2C).

### Construction of Differentially Expressed Immune-Related Long Non-coding Ribonucleic Acid Pairs and Prognosis Signature

**FIGURE 3:**
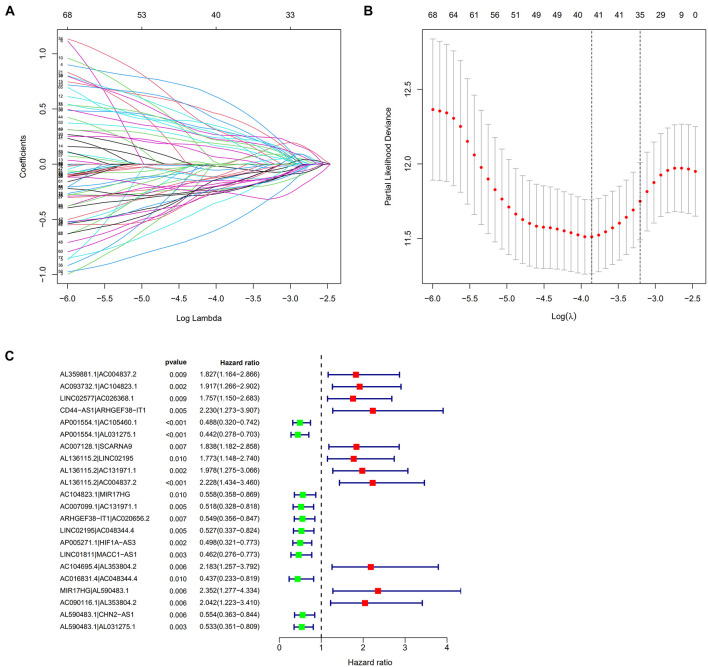
**(A,B)** 22 lncRNA pairs were screened out using Lasso regression analysis. **(C)** A forest map showed 22 DEirlncRNA pairs identified by Cox proportional hazard regression in the stepwise method.

**FIGURE 4:**
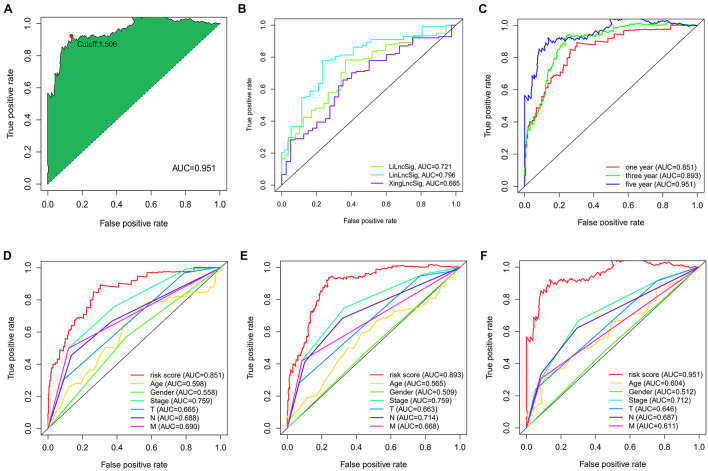
**(A)** The akaike information criterion (AIC) value was calculated to identify the best cut-off point to divide patients into high-risk and low-risk groups. **(B)** The AUC value was much higher than the other three lncRNA-based signatures of colon cancer patients from three other studies. **(C)** The AUC for 1-, 3- and 5-year survival were 0.851, 0.893, and 0.951, respectively. **(D–F)** The AUC of other clinical-pathological features were also presented, and when compared with the constructed signature, the risk score had much better efficiency for predicting 1-, 3- and 5-year survival than the other variables in COAD patients.

**FIGURE 5:**
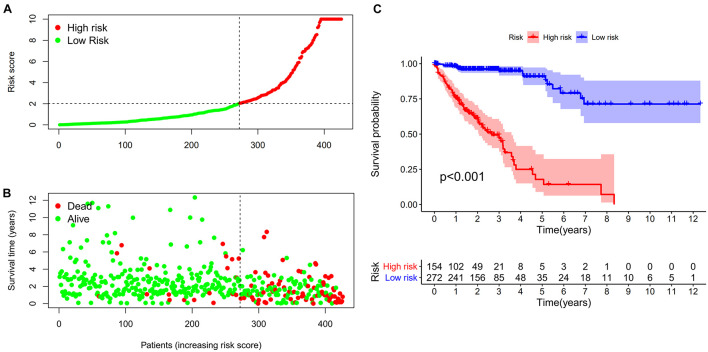
**(A,B)** Risk scores **(A)** and survival outcome **(B)** of each case are shown. **(C)** Patients in the low-risk group experienced a longer survival time tested by the Kaplan-Meier (KM) test.

**FIGURE 6:**
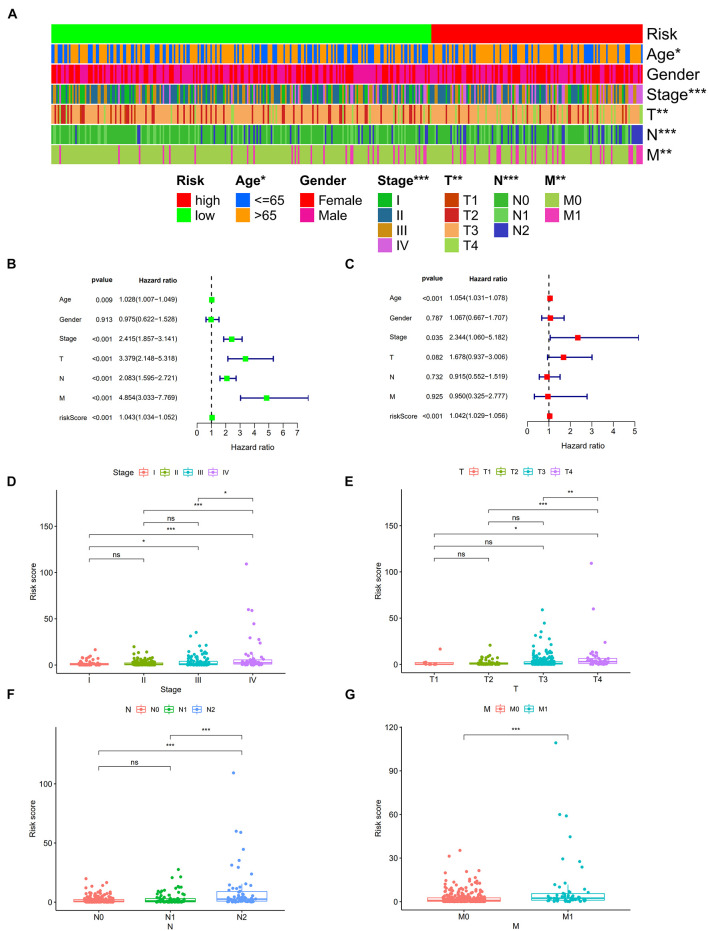
Strip chart **(A)** showed that age, clinical stage, tumor infiltration depth, lymph node metastasis, and distant metastasis were significantly associated with the risk score. Forest plots of univariate **(B)** and multivariate **(C)** Cox regression analyses in colon cancer. Scatters diagram also showed that **(D)** clinical stage, **(E)** tumor infiltration depth, **(F)** lymph node metastasis, and **(G)** distant metastasis status significantly correlated with the risk score. *p < 0.05; **p < 0.01; and ***p < 0.001.

**FIGURE 7:**
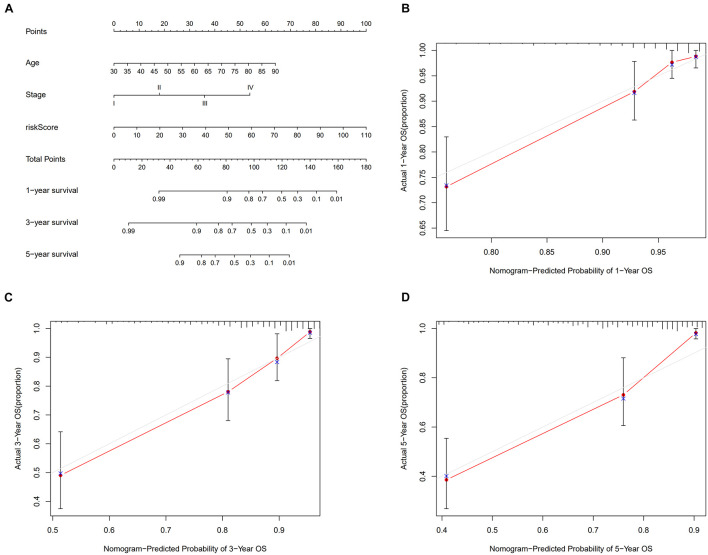
Nomogram model **(A)** to predict 1-, 3-, and 5-year survival rates of colon cancer cases. Calibration graphs indicated that predicted 1- **(B)**, 3- **(C)** and 5- **(D)** year survival rates were close to the actual survival rates.

A total of 2,720 valid lncRNA pairs were constructed based on the 90 DElncRNAs and 22 were screened out using LASSO regression analysis (Figures 3A,B). As shown in Figure 3C, all the 22 lncRNA pairs were significant in univariate Cox regression analysis (*p* < 0.05). The risk score was defined as $\sum_{i=1}^{\text{n}}\beta \,i^{*}\,\lambda \,i$. Then, 5-year AUC for ROC curves of the 22 lncRNA pairs were calculated. The maximum AUC value of the signature to predict 5-year survival was 0.951; the AIC value was also calculated to identify the ideal cut-off point to divide patients into high-risk and low-risk groups (Figure 4A). As shown in Figure 4B, the AUC value of the signature obtained in this study was much higher than that of three other lncRNA-based signatures of patients with colon cancer from three other studies, including LiLncSig (Li Z. et al., 2020) (AUC = 0.721), LinLncSig (Lin et al., 2020) (AUC = 0.796), and XingLncSig (Xing et al., 2018) (AUC = 0.665). The AUC for 1-, 3-, and 5-year survival were 0.851, 0.893, and 0.951, respectively (Figure 4C). AUC of other clinical-pathological features were also presented. In the constructed signature, risk score had a higher efficiency for predicting 1-, 3-, and 5-year survival than other variables in COAD patients (Figures 4D–F). Based on the risk score, patients were divided into high-risk and low-risk groups using the calculated cut-off point (Figure 5A), and patients with higher risk scores had a higher risk of mortality (Figure 5B). As seen in Figure 5C, survival curve was plotted to show the survival differences of COAD patients in the two groups. Patients in the high-risk group had a significantly lower probability of survival than those in the low-risk group (*p* < 0.001). Chi-square tests were performed to investigate the relationship between risk score and other clinical-pathological features. A heatmap was plotted, showing that age, clinical stage, T stage, N stage, and M stage were significantly related to the risk score (Figure 6A). Univariate and multivariate Cox regression analyses were performed to identify prognosis-related factors in COAD patients (Figures 6B,C). Factors with *p* value < 0.05 in the univariate analysis were included in the multivariate analysis. Forest maps showed that age (*p* < 0.001, HR = 1.054, 95% CI [1.031–1.078]), stage (*p* = 0.035, HR = 2.344, 95% CI [1.060–5.182]), and the risk score (*p* < 0.001, HR = 1.042, 95% CI [1.029–1.056]) were still significant after multivariate analysis. Therefore, the risk score was independently associated with the prognosis of COAD patients. Wilcoxon signed-rank test showed that clinical stage (Figure 6D), T stage (Figure 6E), N stage (Figure 6F), and M stage (Figure 6G) were significantly related to the calculated risk score. To better predict 1-, 3-, and 5-year survival rates of COAD cases, a nomogram model was constructed based on the results of univariate and multivariate Cox regression analyses (Figure 7A). Age, clinical stage, and risk score were included in the nomogram model. Accordingly, a 60-year-old patient with stage IV colon cancer and risk score of 45 has an estimated 5-year survival rate of less than 10%. Moreover, calibration plots depicting the differences between nomogram-predicted and actual survival probabilities of COAD patients showed that the predicted 1-, 3-, and 5-year survival probabilities were close to the actual survival probabilities (Figures 7B–D), indicating that this nomogram model accurately predicted survival of COAD patients.

### Evaluation of the Relationship Between Risk Score and Immune Cell Infiltration Status

**FIGURE 8:**
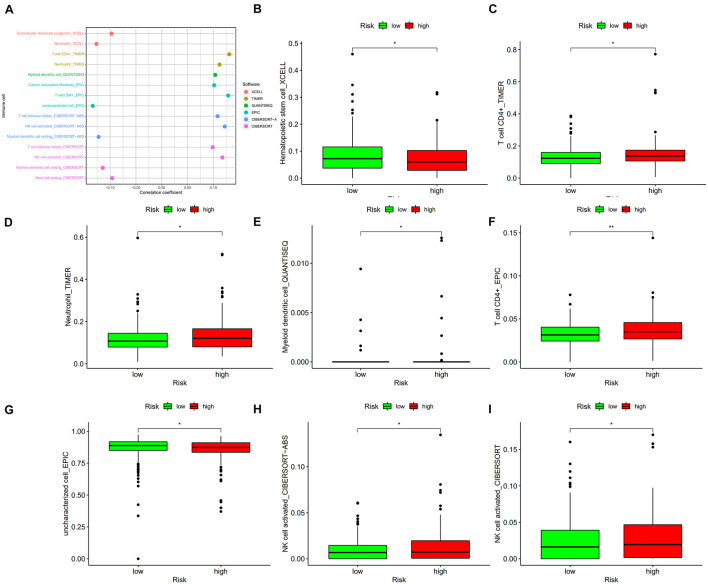
**(A)** The risk score correlated with the presence of many kinds of immune cells, including granulocyte-monocyte progenitors, neutrophils, CD4+ T cells, myeloid dendritic cells, cancer-associated fibroblasts, and activated NK cells, among others. A high risk score was positively associated with the presence of tumor infiltrating immune cells, including CD4+ T cells **(C,F)**, neutrophils **(D)**, and activated NK cells **(H,I)**, as well as was negatively associated with the presence of hematopoietic stem cells **(B)**, myeloid dendritic cells **(E)**, and uncharacterized cells **(G)**. *p < 0.05; **p < 0.01.

To better understand the correlation between risk score and tumor immune microenvironment, the Spearman correlation and Wilcoxon signed-rank tests were performed. As Figure 8A shows, the risk score correlated with many types of immune cells, including granulocyte-monocyte progenitors, neutrophils, CD4+ T cells, myeloid dendritic cells, cancer associated fibroblasts, and activated natural killer (NK) cells. High risk score was positively associated with tumor infiltrating immune cells including CD4+ T cells (Figures 8C,F), neutrophils (Figure 8D), and activated NK cells (Figures 8H,I), and negatively associated with hematopoietic stem cells (Figure 8B), myeloid dendritic cells (Figure 8E), and uncharacterized cells (Figure 8G).

### Expression of Immune Checkpoint Genes in High-Risk and Low-Risk Groups

**FIGURE 9:**
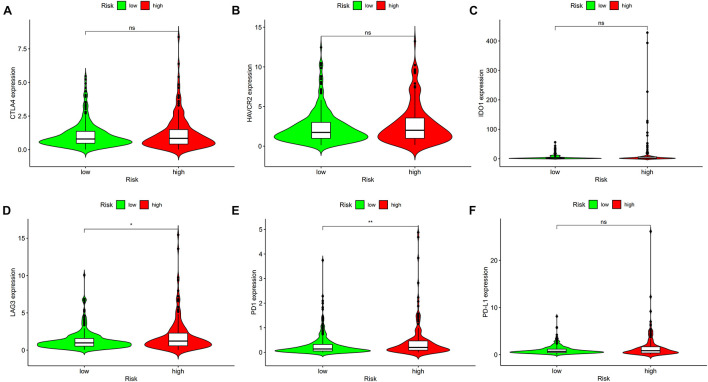
High risk scores were positively correlated with upregulated **(D)** lymphocyte-activation gene 3 (LAG3) and **(E)** programmed cell death protein 1 (PD1) levels, whereas the other plots showed no statistical difference in patients with colon cancer **(A–C,F)**. *p < 0.05; **p < 0.01.

To use the risk score to predict potential checkpoint blockade therapy, violin plots were drawn to show the differences of immune checkpoint gene expression in high-risk and low-risk groups. LAG3 (Figure 9D, *p* < 0.05) and PD-1 (Figure 9E, *p* < 0.01) expressions were significantly different in the two groups, whereas CTLA4 (Figure 9A, *p* > 0.05), HAVCR2 (Figure 9B, *p* > 0.05), IDO1 (Figure 9C, *p* > 0.05), and PD-L1 (Figure 9F, *p* > 0.05) expressions showed no significant difference between the groups.

### Using the Risk Score to Predict Chemosensitivity of COAD Patients

**FIGURE 10:**
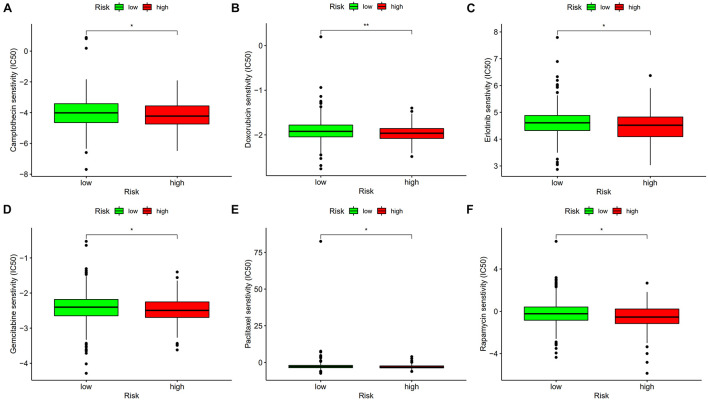
Lower IC50 of camptothecin **(A)**, doxorubicin **(B)**, erlotinib **(C)**, gemcitabine **(D)**, paclitaxel **(E)**, and rapamycin **(F)** were associated with a higher risk score. *p < 0.05; **p < 0.01.

The differences in chemosensitivity evaluated using IC50 values in high-risk and low-risk groups were analyzed using Wilcoxon signed-rank test. The results indicated that lower IC50 values of camptothecin, doxorubicin, erlotinib, gemcitabine, paclitaxel, and rapamycin were associated with higher risk scores (Figure 10). These results might provide reference for clinical treatment of COAD.

### Validating Expression Levels of LINC02195 and SCARNA9 *via* Quantitative Real-Time Polymerase Chain Reaction

**FIGURE 11:**
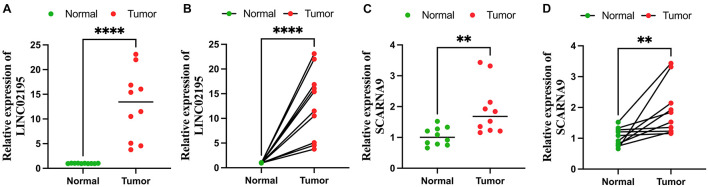
Quantitative real-time polymerase chain reaction (qRT-PCR) analyses of LINC02195 **(A,B)** and SCARNA9 **(C,D)** expression in 10 pairs of colon cancer tissues and adjacent non-cancer tissues. **p < 0.01; and ****p < 0.0001.

To explore the expression levels of LINC02195 and SCARNA9, these lncRNAs were tested in colon cancer and adjacent non-cancer tissues using qRT-PCR method. As Figure 11 shows, the expression levels of LINC02195 and SCARNA9 in colon cancer were significantly higher than that in adjacent non-cancer tissues.

## Discussion

Recent studies have presented signatures for cancer diagnosis and survival prediction based on the specific expression levels of coding genes or noncoding RNAs (Liu et al., 2021; Wang R. et al., 2021; Wang X. et al., 2021; Wu et al., 2021), which required certain testing methods. In this study, irlncRNA pairs were constructed and used for the development of prognosis signature. The combination of two lncRNAs did not require the exact expression quantities to be measured by certain methods, making it much more feasible and convenient in clinical use.

First, the expression and clinical data were retrieved from TCGA database. Differential co-expression analysis was performed to identify DEirlncRNAs and GO and KEGG analyses were performed to explore relevant pathways and molecular biological functions. The results suggested that DEirlncRNAs were related to the biological functions of the organization of cellular and noncellular components. Malignant tumors are surrounded by extracellular matrix and stromal cells and these cellular and non-cellular components build up the tumor microenvironment (Wang et al., 2017). The interactions between tumor microenvironment and cancer cells have great importance in cancer progression and metastasis (Joyce and Pollard, 2009; Quail and Joyce, 2013). Results showed that DEirlncRNAs participated in the pathways of protein digestion and absorption, WNT signaling, nitrogen metabolism, and regulating pluripotency of stem cells. The WNT signaling pathway is related to several cancer types, especially colorectal cancer (CRC; Cancer Genome Atlas Network, 2012). Wnt-β-catenin signal activation leads to the accumulation of β-catenin in the nucleus, which has been detected in over 80% of CRC tumor tissues (Wanitsuwan et al., 2008). In addition, high levels of nuclear β-catenin are associated with poor prognosis in patients with CRC (Baldus et al., 2004). A recent study indicated that nitrogen metabolism in was changed in various types of cancer, which was detectable in body fluids and might cause new mutations in cancer tissues (Lee et al., 2018). Crespo et al. (2018) suggested that colonic organoids from human-induced pluripotent stem cells can be used for modeling CRC.

A total of 2,720 valid lncRNA pairs were constructed using 90 DElncRNAs. To explore the impact of DElncRNA pairs on prognosis in colon cancer patients, 22 prognosis-related DElncRNA pairs were identified using LASSO regression analysis and Cox regression analysis. Some of the DEirlncRNAs used for modeling have already been demonstrated to play an important role in CRC and other types of malignant tumors. Anirban et al. (Li H. et al., 2020) demonstrated that LINC02195 is a regulator of histocompatibility complex class I molecules and a prognosis biomarker for head and neck squamous cell carcinoma. Wang et al. (2020) showed that SCARNA9 is associated with the prognosis of patients with endometrial cancer. Xu et al. (2019) suggested that MIR17HG is an immune-related lncRNA, as it was upregulated in CRC tissues compared with normal tissues. Moreover, MIR17HG also contributed to tumorigenesis and metastasis in CRC cells both *in vitro* and *in vivo*. Cao et al. (2019) also showed that MIR17HG is upregulated in glioma tissues and cell lines and that downregulation of MIR17HG is related to inhibition of glioma cell progression. Therefore, the constructed signature can identify new biomarkers for further studies.

Subsequently, every AUC value was calculated to construct a signature with the maximum AUC value. The 1-, 3- and 5-year AUCs of the prognosis signature were also compared with other clinical variables. AIC value was calculated to find the ideal cut-off point to divide patients into high- and low-risk groups. KM curve showed that survival rates of low-risk patients were much higher than those of high-risk patients. To better understand the utility of clinical variables and risk score on predicting patient outcomes, univariate and multivariate Cox analyses were performed. These results showed that the risk score remained significant after these analyses, indicating that the calculated risk score was an independent predictor of patient prognosis. The relationship between the risk score and other clinical features was also determined. The results suggested that the risk score correlated with tumor stage, tumor infiltration depth, lymph mode metastasis, and distant metastasis, indicating that the risk score might be related to the development and migration of colon cancer. Nomogram is a prediction tool in oncology, especially for cancer prognosis (Iasonos et al., 2008; Balachandran et al., 2015). A nomogram model was established to visualize the effects of clinical features and risk score on 1-, 3- and 5-year survival probabilities of patients. Calibration graphs showed that the nomogram-predicted survival rates were close to actual survival rates, indicating that the nomogram model had high prediction efficiency.

Tumor-infiltrating immune cells affect the response to tumor anti-checkpoint blockades. Tumor-infiltrating CD4+ T cells upregulated PD-1, cytotoxic T-lymphocyte-associated protein-4 (CTLA-4), T cell immunoglobulin and mucin domain-3 (TIM-3) and LAG-3 (Toor et al., 2019). Six common methods were used to evaluate the relationship between tumor infiltration immune cells and risk score, including XCELL (Aran et al., 2017), TIMER (Li et al., 2017), QUANTISEQ (Finotello et al., 2019), EPIC (Van Veldhoven et al., 2011), CIBERSORT-A (Tamminga et al., 2020), and CIBERSORT (Newman et al., 2015). The results revealed that the risk score was positively related to CD4+ T cells, neutrophils, and activated NK cells, and negatively related to hematopoietic stem cells and myeloid dendritic cells. A previous study showed that immune scores based on immune genomic analysis can indicate the therapeutic benefits of immunotherapy and chemotherapy (Dai et al., 2020). To predict potential checkpoint blockade therapy, the relationship between risk score and immune checkpoint gene expression was explored and the results showed that PD-1 and LAG-3 expressions were significantly different in high- and low-risk groups. Anti-PD-1 inhibitor is effective in treating patients with dMMR/MSI-H mCRC (He et al., 2020). Xiao et al. (Xiao and Freeman, 2015) suggested that LAG-3 creates an immunosuppressive microenvironment in MSI-H CRC, possibly helping MSI-H tumors escape immune destruction by infiltrating immune cells. Our study also revealed that the risk score was associated with sensitivity to chemotherapeutics such as camptothecin, doxorubicin, erlotinib, gemcitabine, paclitaxel, and rapamycin. The findings our study can be applied for guiding clinical immunotherapy and chemotherapy in patients with colon cancer. Among lncRNAs that were used to construct the signature, LINC02195 and SCARNA9 were found to be assiciated with neck squamous cell carcinoma and endometrial cancer in previous researches (Li H. et al., 2020; Wang et al., 2020), however, the effects of these two irlncRNAs on colon cancer remains unknown. We further validated the expression levels of LINC02195 and SCARNA9 using qRT-PCR method, the results showed that LINC02195 and SCARNA9 were significantly upregulated in colon cancer compared with adjacent non-cancer tissues, indicating that the two irlncRNAs may be potential biomarkers for diagnosis and therapy in colon cancer.

However, the study has some shortcomings and limitations. All data in our study were downloaded from TCGA database because we could not find other datasets that simultaneously included lncRNA expression levels, clinicopathological characteristics, and survival outcomes for patients with colon cancer. The individual data source might result in unreliable results. Subsequent molecular biological experiments are needed to further examine the function of DEirlncRNAs in colon cancer development and to better understand carcinogenic mechanisms. In addition, clinical cases are required in further studies to increase stability and the predictive ability of our established signature.

## Conclusion

In conclusion, our analysis of lncRNA expression profiles and clinical features identified DEirlncRNAs in colon cancer. LncRNA pairs were constructed and used for the development of prognosis signature, which did not require the exact expression quantities tested by certain methods. The constructed signature could effectively evaluate the prognosis of patients with colon cancer and guide clinical therapy. Additional studies are needed to validate the findings of this study and provide a basis for individualized treatment of patients with colon cancer.

## Data Availability Statement

The datasets presented in this study can be found in online repositories. The names of the repository/repositories and accession number(s) can be found in the article/Supplementary Material.

## Ethics Statement

The studies involving human participants were reviewed and approved by the Committee on Medical Ethics of The First Affiliated Hospital of Anhui Medical University. The patients/participants provided their written informed consent to participate in this study.

## Author Contributions

XW and KC are responsible for writing and submitting the manuscript. ZW, BC, and YX are responsible for data collection and analysis. LD and TB are responsible for the production of pictures. WY and WC are responsible for the ideas and guidance. All authors have read and approved the final manuscript.

## Conflict of Interest

The authors declare that the research was conducted in the absence of any commercial or financial relationships that could be construed as a potential conflict of interest.

## Publisher’s Note

All claims expressed in this article are solely those of the authors and do not necessarily represent those of their affiliated organizations, or those of the publisher, the editors and the reviewers. Any product that may be evaluated in this article, or claim that may be made by its manufacturer, is not guaranteed or endorsed by the publisher.

## Footnotes

1
https://portal.gdc.cancer.gov

2
http://asia.ensembl.org

3
http://www.immport.org

## Supplementary Material

Supplementary Table 1 Clinicopathologic characteristics of TCGA colon cancer patients.

Supplementary Table 2 List of 90 DEirlncRNAs.

Supplementary Table 3 Univariate Cox regression analysis of the 22 identified lncRNA pairs.

Supplementary Table 4 Univariate Cox regression analysis of risk score and other clinical features.

Supplementary Table 5 Multivariate Cox regression analysis of risk score and other clinical features.

Supplementary Table 6 The correlation between risk score and immune infiltration of cells *via* the Spearman correlation method.

Supplementary Table 7 Raw data of qRT-PCR.
