# Supplementary material for: Using Immune-Related Long Non-coding Ribonucleic Acids to Develop a Novel Prognosis Signature and Predict the Immune Landscape of Colon Cancer
Source: Front Cell Dev Biol. 2021 Sep 30;9:750709. doi: 10.3389/fcell.2021.750709 (PMC8514752; doi:10.3389/fcell.2021.750709)
Supplement: Supplementary Table 1 — Clinicopathologic characteristics of TCGA colon cancer patients. [file Table_1.DOC]

**Supplementary Table 1** Clinicopathologic characteristics of TCGA colon cancer patients

| **Clinicopathologic features** |  | **Total (459)** | **%** |
| --- | --- | --- | --- |
| **age at diagnosis (year)** |  | 68 (31-90) |  |
| **sex** | male  female | 243  216 | 52.9  47.1 |
| **race** | asian  black or african american  white | 11  233  213 | 2.5  51.0  46.5 |
| **stage** | stage I  stage II  stage III  stage IV | 68  166  146  65 | 15.3  37.3  32.8  14.6 |
| **tumor (T)** | T1  T2  T3  T4 | 11  78  313  56 | 2.5  17.0  68.2  12.3 |
| **node (N)** | positive  negative | 189  270 | 41.2  58.8 |
| **metastasis (M)**  **living status** | positive  negative  alive  dead | 67  341  357  102 | 16.4  83.6  77.8  22.2 |
